# Phospholipid transfer protein and **a**lpha-1 antitrypsin regulate Hck kinase activity during neutrophil degranulation

**DOI:** 10.1038/s41598-018-33851-8

**Published:** 2018-10-18

**Authors:** Pius Ochieng, Sridesh Nath, Reane Macarulay, Edward Eden, Abdoulaye Dabo, Michael Campos, Xian-Cheng Jiang, Robert F. Foronjy, Patrick Geraghty

**Affiliations:** 1 Pulmonary, Critical Care and Sleep Division, Geisinger Medical Center, Danville, PA 17822 USA; 2 Department of Medicine, State University of New York Downstate Medical Center, Brooklyn, NY 11203 USA; 3 Trinity College, Dublin 2, Ireland; 4 Division of Pulmonary and Critical Care Medicine, Mount Sinai St Luke’s Hospital, New York, NY 10025 USA; 5 Department of Cell Biology, State University of New York Downstate Medical Center, Brooklyn, NY 11203 USA; 6 University of Miami Miller School of Medicine, Miami, Florida, FL 33136 USA

## Abstract

Excessive neutrophil degranulation is a common feature of many inflammatory disorders, including alpha-1 antitrypsin (AAT) deficiency. Our group has demonstrated that phospholipid transfer protein (PLTP) prevents neutrophil degranulation but serine proteases, which AAT inhibits, cleave PLTP in diseased airways. We propose to identify if airway PLTP activity can be restored by AAT augmentation therapy and how PLTP subdues degranulation of neutrophils in AAT deficient subjects. Airway PLTP activity was lower in AAT deficient patients but elevated in the airways of patients on augmentation therapy. Functional AAT protein (from PiMM homozygotes) prevented PLTP cleavage unlike its mutated ZZ variant (PiZZ). PLTP lowered leukotriene B4 induced degranulation of primary, secondary and tertiary granules from neutrophils from both groups (n = 14/group). Neutrophils isolated from *Pltp* knockout mice have enhance neutrophil degranulation. Both AAT and PLTP reduced neutrophil degranulation and superoxide production, possibly though their inhibition of the Src tyrosine kinase, Hck. Src kinase inhibitors saracatinib and dasatinib reduced neutrophil degranulation and superoxide production. Therefore, AAT protects PLTP from proteolytic cleavage and both AAT and PLTP mediate degranulation, possibly via Hck tyrosine kinase inhibition. Deficiency of AAT could contribute to reduced lung PLTP activity and elevated neutrophil signaling associated with lung disease.

## Introduction

Alpha-1 antitrypsin (AAT) deficiency is an inherited genetic disorder and is the most common genetic factor associated with chronic obstructive pulmonary disease^1^. AAT is an abundant circulating serine protease inhibitor that is primarily known to counter the activity of neutrophil elastase (NE)^2^. Currently, AAT deficiency is treated with AAT augmentation therapy, with patients receiving well tolerated weekly infusions of plasma-purified AAT that increase circulating and airway levels of AAT^3^. Recently, multiple extracellular roles of AAT have emerged^4,5^, including activation of phosphatases^6^, inhibition of caspase activity^7^ and nitric oxide production^8^, subduing HIV type 1 infectivity^9^ and ER stress responses^10,11^, minimizing epithelial barrier damage and IL-8 mediated neutrophil chemotaxis^12^. AAT also regulates neutrophil degranulation, as neutrophils from AAT deficient subjects are more sensitive to degranulation^13,14^. Our group recently identified another important role of AAT, with AAT protecting against the degradation of an important anti-inflammatory protein, phospholipid transporter protein (PLTP)^15^. Identifying how AAT and PLTP alter neutrophilic inflammation could provide novel new insights into the pathogenesis of AAT deficiency.

PLTP is a glycoprotein that is secreted into the plasma^16^ and other fluid compartments^17^ where it regulates multiple biological processes^18,19^. Increased PLTP expression is observed in atherosclerotic plaques^20,21^, diabetes^22^, obesity^23^ and in the lung tissue of COPD patients^24^. However, the increased lung PLTP gene expression observed in COPD^24^ occurs in combination with reduced airway PLTP activity, due to serine protease cleavage of PLTP^15^. Functionally, PLTP is primarily known to shuttle phospholipids to lipoprotein particles^19^, which contributes to the formation of smaller lipoprotein remnants, such as LDL and HDL^25^. PLTP also promotes the uptake and transport of cholesterol from peripheral cells to the liver^26^. Additionally, PLTP has anti-inflammatory effects, via ABCA1 signaling, to induce STAT3 signaling^27^ and block NF-κB activation and cytokine expression^28^. Loss of *Pltp* leads to increased septic shock death in mice^29^. PLTP also reduces LTB_4_ induced degranulation in human neutrophils^15^ but the mode of its inhibitory neutrophil degranulation action is not known. Therefore, PLTP’s anti-inflammatory potential and signaling transduction in reducing inflammation associated with lung diseases require further investigations.

This study aimed to identify whether AAT therapy prevents loss of PLTP activity and how PLTP could alter neutrophil degranulation signaling. Here, we demonstrate that PLTP activity in bronchoalveolar lavage fluid (BALF) from AAT deficient subjects is decreased due to PLTP cleavage in the airways and intravenous infusion of the non-mutated form of the AAT protein can prevent this cleavage within the airways. Furthermore, PLTP subdued superoxide production and degranulation in neutrophils isolated from AAT competent and deficient individuals. Neutrophils from *Pltp* deficient mice were more sensitive to stimuli triggering degranulation than neutrophils from wild type animals, but both genotypes responded to AAT treatment. Both AAT and PLTP inhibited the phosphorylation of Src kinases at the tyrosine 416 site^30^ and the tyrosine kinase activity of the Src kinase, Hck, a known regulator of neutrophil degranulation responses to fMLP^31,32^. Thus, these findings establish that AAT and PLTP can lower neutrophil degranulation responses separately of one another possibly by targeting the activity of Hck.

## Results

### Airway PLTP activity is enhanced in BALF of AAT deficient subjects on AAT augmentation therapy

**Figure 1:**
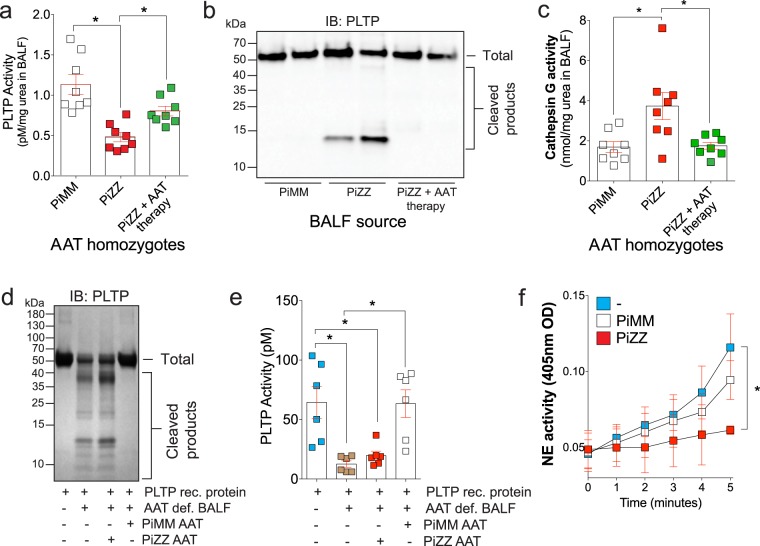
Lung extracellular PLTP activity is enhanced in BALF of AAT deficient subjects on AAT augmentation therapy. (**a**) PLTP activity was measured in lung BALF from age-matched PiMM, PiZZ subjects and PiZZ subjects on AAT augmentation therapy. (**b**) PLTP immunoblot from concentrated (10-fold) BALF from PiMM, PiZZ subjects and PiZZ subjects on AAT augmentation therapy. (**c**) BALF cathepsin G activity levels were determined and corrected to urea concentrations. (**d**) BALF from AAT deficient subjects was incubated with AAT isolated from either PiMM or PiZZ plasma for one hour followed by incubation with recombinant PLTP for 24 hours and PLTP immunoblots were performed and (**e**) PLTP activity recorded. (**f**) Antiprotease activity assays were performed to determine the activity of the PiMM and PiZZ forms of AAT isolated from subjects (n = 6/group). Graphs are represented as mean ± S.E.M. *Denotes a p value < 0.05, when comparing both treatments connected by a line, determined by 2-way ANOVA with Tukey’s post hoc test.

Since the major site of PLTP activity is extracellular^33^ and PLTP BALF activity is decreased in COPD subjects^15^, we investigate the status of lung PLTP activity in AAT deficient COPD subjects. PLTP activity was significantly decreased in the BALF from AAT deficient subjects (from PiZZ homozygote subjects), compared to healthy controls (PiMM, Fig. 1a). Importantly, PLTP activity was higher in AAT deficient subjects on AAT augmentation therapy compared to patients not on therapy (Fig. 1a). PLTP immunoblotting on BALF protein demonstrated that PLTP undergoes extracellular degradation in the diseased lung but augmentation therapy protects PLTP from cleavage (Fig. 1b). Serine proteases cleave PLTP^15^ and we observed elevated levels of a serine protease, cathepsin G, in the airways of AAT deficient subjects not on AAT augmentation therapy (Fig. 1c). To investigate whether PLTP protein undergoes degradation in the presence of disease BALF, recombinant PLTP (rPLTP) was incubated with BALF from AAT deficient subjects not on augmentation therapy for 24 hours at 37 °C and analyzed by PLTP immunoblotting (d. 1d). Breakdown products of rPLTP was observed after incubation with BALF from diseased lungs not on AAT augmentation therapy (Fig. 1d). To identify whether functional (PiMM) or a mutated form of (PiZZ) AAT could prevent PLTP cleavage, AAT was pre-incubated with BALF from AAT deficient subjects not on augmentation therapy, before adding rPLTP. After incubation at 37 °C for 24 hours, samples were analyzed by PLTP immunoblotting. Pre-incubation with the PiMM AAT prevented disease BALF cleavage of rPLTP (Fig. 1d), which coincided with PLTP activity similar to controls (Fig. 1e). The PiZZ form of AAT could not prevent rPLTP cleavage or loss of activity (Fig. 1d,e). Antiprotease potential of each form of AAT, PiMM and PiZZ, was confirmed in a NE activity assay with PiZZ observed to have less inhibitory potential/antiprotease activity (Fig. 1f). Therefore, proteases present in AAT functionally deficient BALF cleave extracellular PLTP and AAT augmentation therapy reverses this loss of PLTP activity.

### PLTP lowers degranulation rate in neutrophils from AAT competent or deficient subjects

**Figure 2:**
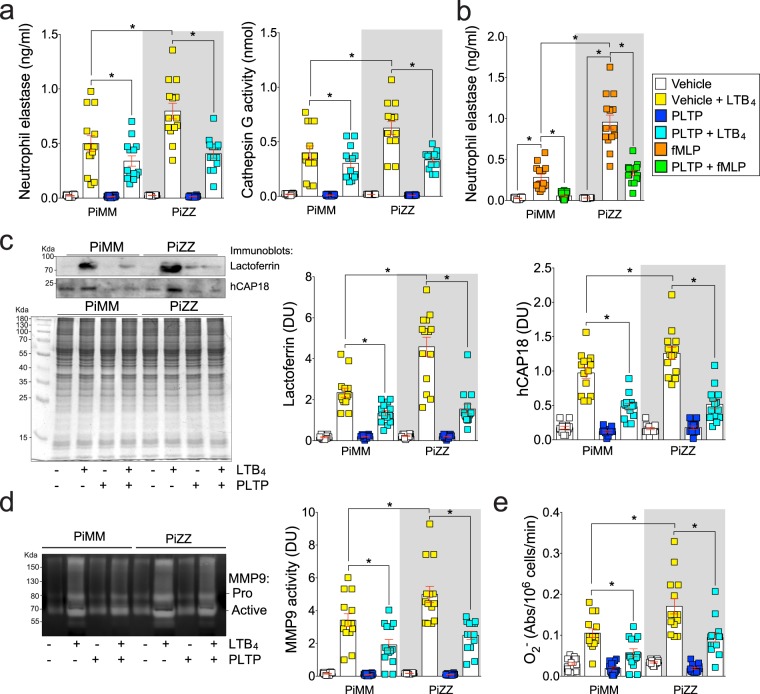
PLTP reduces release of neutrophil granules and activation in AAT deficient subjects. Neutrophils from PiMM and PiZZ subjects were exposed to LTB_4_ (**a**,**c**–**e**) or fMLP (**b**) at 37 °C for 30 minutes, following stimulation with vehicle (albumin) or PLTP protein. Cell-free supernatants were collected and (**a,b**) primary (NE by ELISA and cathepsin G by substrate activity assay), (**c**) secondary (hCAP-18 and lactoferrin by immunoblots) and (**d**) tertiary (MMP9 by zymography) granules were evaluated. NE degranulation was recorded in fMLP and PLTP stimulated neutrophils to determine if PLTP prevent degranulation in more than one stimulus. (**b**) The use of equal cell numbers in each reaction is demonstrated by the identical electrophoretic profile of whole cell lysates in the Coomassie blue stained gels (loading control; bottom panel). Densitometry units (DU) of the expression levels of each protein were quantified. (**e**) A reduced cytochrome c assay was used to determine production of O_2_^−^ by neutrophils. N = 14 subjects per group. Each measurement is the mean ± SEM. *Denotes a p value < 0.05, when comparing both treatments connected by a line, by 2-way ANOVA with Tukey’s post hoc test.

To demonstrate if PLTP could prevent granule release and alter degranulation in neutrophils from AAT deficient subjects, neutrophils were isolated from peripheral blood and exposed to PLTP prior to LTB_4_ stimulation (Fig. 2). Similar to other studies^13,14^, neutrophils from AAT deficient subjects degranulated more compared to neutrophils from AAT competent subjects, with elevated primary (Fig. 2a, NE concentration and cathepsin G activity for LTB_4_ and Fig. 2b, NE for N-Formylmethionyl-leucyl-phenylalanine (fMLP)), secondary (Fig. 2c; lactoferrin and hCAP18) and tertiary (Fig. 2d; MMP9) granules released at a higher rate. Importantly, pretreatment with PLTP reduced degranulation in neutrophils from both PiMM and PiZZ individuals from each granule (Fig. 2). PLTP was also observed to reduce superoxide (O_2_^−^) production both in PiMM and PiZZ subjects (Fig. 2e). Therefore, PLTP can reduce the priming of neutrophils and secretion of neutrophil granules both in PiMM and PiZZ subjects.

### Loss of PLTP expression enhances degranulation and superoxide production in mouse neutrophils

**Figure 3:**
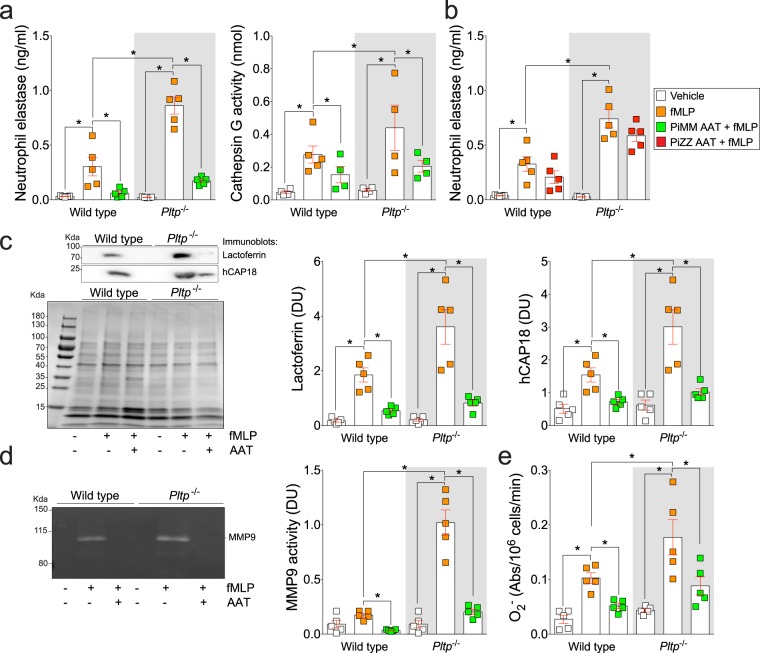
Loss of PLTP expression enhances neutrophil degranulation and superoxide production. Neutrophils were collected from C57BL/6 and *Pltp*^−/−^ mice. Neutrophils were exposed to AAT (PiMM: **a,c**–**e** and PiZZ: **b**) prior to fMLP at 37 °C for 30 minutes. Cell-free supernatants were collected and (**a**,**b**) primary (NE by ELISA and cathepsin G by substrate activity assay), (**c**) secondary (hCAP-18 and lactoferrin by immunoblots) and (**d**) tertiary (MMP9 by zymography) granules were evaluated. DU of the expression levels of each protein were quantified. (**b**) NE degranulation was recorded in fMLP and PiZZ AAT stimulated neutrophils to determine if PiZZ prevent degranulation. (**e**) A reduced cytochrome c assay was used to determine production of O_2_^−^ by neutrophils. N = 5 animal per group. Each measurement is the mean ± SEM. *Denotes a p value < 0.05, when comparing both treatments connected by a line, determined by ANOVA with Tukey’s post hoc test.

Since extracellular PLTP can reduce neutrophil degranulation, we investigated whether PLTP expression alters neutrophil degranulation potential. To test this hypothesis, neutrophils from *Pltp*^−/−^ mice were isolated from bone marrow and stimulated with fMLP. Compared to wild type mice, neutrophils from *Pltp*^−/−^ mice release greater concentrations of granules, with elevated primary (Fig. 3a; NE and cathepsin G and Fig. 3b for NE), secondary (Fig. 3c; lactoferrin and hCAP18) and tertiary (Fig. 3d; MMP9) observed. Pretreating neutrophils with AAT protein from PiMM subjects reduced fMLP induced degranulation from both wild type and *Pltp*^−/−^ neutrophils (Fig. 3). AAT from PiZZ subjects did not prevent fMLP induced NE degranulation (Fig. 3b). PLTP expression was also observed to alter O_2_^−^ production that was reversed upon AAT stimulation (Fig. 3e). Therefore, loss of PLTP expression enhances neutrophil degranulation.

### Neutrophils from *Pltp* deficient mice has elevated activity of Src kinase Hck

**Figure 4:**
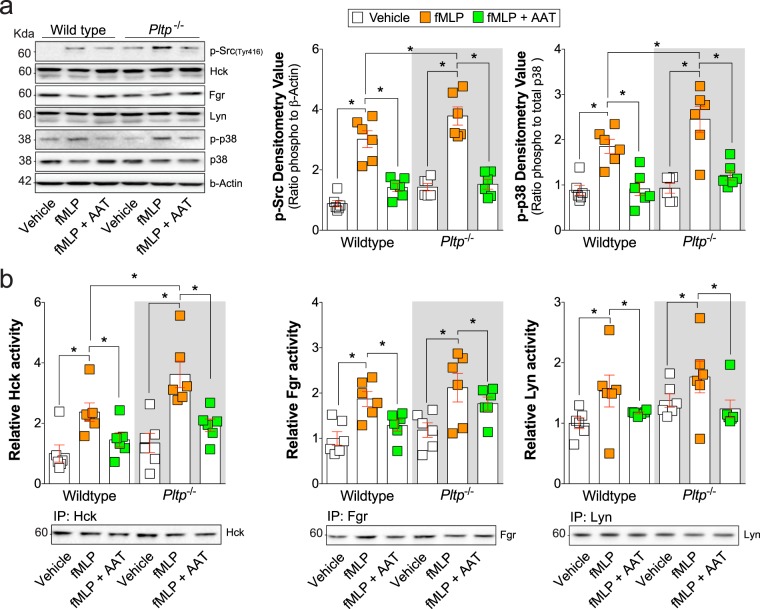
Neutrophils from *Pltp* deficient mice has elevated tyrosine kinase activity of Hck. Neutrophils were collected from C57BL/6 and *Pltp*^−/−^ mice. Neutrophils were exposed to AAT prior to fMLP at 37 °C for 5 minutes. Immunoblots were performed for (**a**) phosphorylated Src kinase family (Tyr416) and total Hck, Fgr, Lyn, phosphorylated and total p38, and β-Actin. Densitometry was performed for p-p38 and p-Src. (**b**) HCK, Fgr and Lyn were immunoprecipitated and tyrosine kinases activity assays and immunoblots were performed with the IP product. Data is represented as relative activity, corrected to absorbance for the wild type non-treated neutrophil group. N = 6 animal per group. Each measurement is the mean ± SEM. *Denotes a p value < 0.05, when comparing both treatments connected by a line, determined by 2-way ANOVA with Tukey’s post hoc test.

The Src family of tyrosine kinases can regulate the release of granules from fMLP stimulated neutrophils^34^. Here, we investigated whether the activities of Src kinases were contributing to the elevated neutrophil degranulation from *Pltp*^−/−^ mice and determined whether AAT alters fMLP-mediated Src kinase activation in neutrophils. Neutrophils from *Pltp*^−/−^ mice have greater phosphorylation at the 416-tyrosine site with fMLP stimuli compared to wild type neutrophils, which was reduced upon AAT stimuli (Fig. 4a). Treatment with functional AAT directly inhibited p38 phosphorylation upon fMLP stimuli (Fig. 4a). *Pltp* deficiency resulted in elevated p38 phosphorylation that was reversed by AAT (Fig. 4a). These p38 changes (Fig. 4a) coincided with elevated tyrosine kinase activity of Hck that can regulate p38^31^ (Fig. 4b). Immuno-precipitated products demonstrate equal protein loading for each tyrosine target (Fig. 4b).

**Figure 5:**
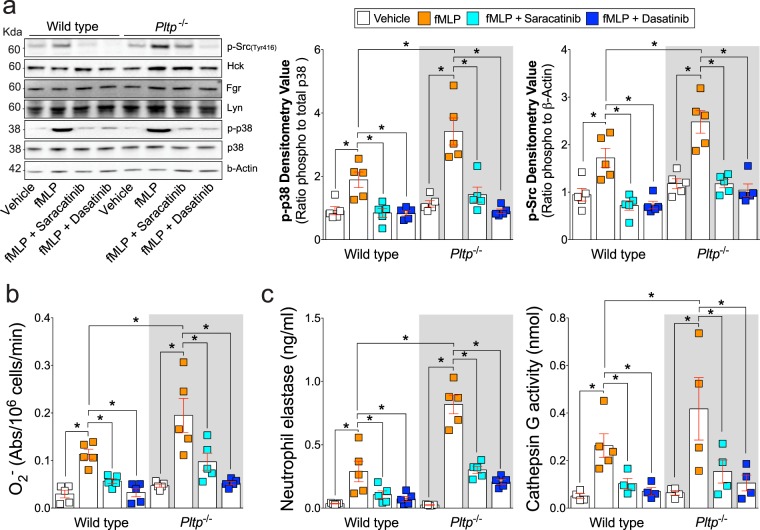
Chemical inhibition of Src tyrosine kinase activity subdues neutrophil degranulation in *Pltp*^−/−^ mice. Neutrophils were collected from C57BL/6 and *Pltp*^−/−^ mice and were exposed to Src inhibitors, saracatinib or dasatinib, prior to fMLP at 37 °C for (**a**) 5 minutes or (b-cu unit3d) 0 minutes. (**b**) Immunoblots were performed for phosphorylated Src kinase family (Tyr416) and total Hck, Fgr, Lyn, phosphorylated and total p38, and β-Actin. Densitometry was performed for p-p38 and p-Src. (**c**) A reduced cytochrome c assay was used to determine production of O_2_^−^ by neutrophils. (**c**) Cell-free supernatants were collected and primary (NE by ELISA and cathepsin G by substrate activity) granules were evaluated. N = 5 animal per group. Each measurement is the mean ± SEM. *Denotes a p value < 0.05, when comparing both treatments connected by a line, determined by 2-way ANOVA with Tukey’s post hoc test.

To determine whether chemical inhibition of Src kinases could reduce neutrophil activation, we investigate neutrophil activation in the presence of the two Src kinase inhibitors saracatinib or dasatinib. Saracatinib inhibits Src and Bcr-Abl tyrosine-kinase activity and dasatinib blocks Src and Bcr-Abl tyrosine-kinases, c-KIT, EPHA2, and PDGFRβ. Both saracatinib or dasatinib treatment reduced fMLP induced Src and p38 activation in neutrophils from both wild type and *Pltp*^−/−^ mice (Fig. 5a). Saracatinib or dasatinib also prevented fMLP induced O_2_^−^ production (Fig. 5b) and degranulation, observed by NE and cathepsin G release (Fig. 5c). These data demonstrate targets of saracatinib or dasatinib, at least partially, regulate neutrophil degranulation and AAT and PLTP both reduced Src kinase activities.

## Discussion

**Figure 6:**
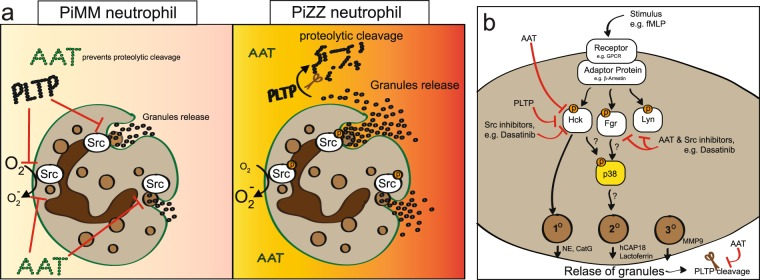
Proposed inhibition of neutrophil degranulation by AAT and PLTP. (**a**) Left panel, functional AAT (PiMM) prevents the cleavage of PLTP by proteases. Both AAT and PLTP prevent Src kinase activation to reduce superoxide production and degranulation. Right panel, AAT deficiency (PiZZ) results in reduced AAT circulation and elevated PLTP cleavage in the lungs. Reduced AAT and cleaved PLTP are less effective at minimizing superoxide production and degranulation in neutrophils. (**b**) Stimulation with fMLP or LTB_4_ induces a signaling cascade leading to Hck activation and granule release. PLTP expression, AAT stimulation or Src inhibitors reduces Hck activation and degranulation. Fgr is known to regulate p38 activation but Hck could also contribute. Release of granulates further enhance PLTP cleavage and AAT complexes to serine proteases, which could further enhance degranulation.

In the present study, we show that PLTP activity is reduced in AAT deficient airways but is similar to normal baseline levels when subjects are receiving AAT therapy. This is likely due to the PiMM form of AAT preventing proteolytic cleavage of PLTP, unlike the mutated PiZZ form. PLTP can also directly prevent neutrophil superoxide production and degranulation from both PiMM and PiZZ donors. Neutrophils from *Pltp*^−/−^ mice degranulate at a greater rate than cells from wild type animals. Similar to wild type mice, neutrophils from *Pltp*^−/−^ mice are sensitive to AAT treatment. Both AAT and PLTP target the deactivation of Src kinase family member, Hck, to reduce neutrophil superoxide production and degranulation (Fig. 6). AAT also prevents activation of other Src kinase family members, Fgr and Lyn. Therefore, this study demonstrates that AAT protects PLTP from degradation and loss of activity, which aids in reducing neutrophil associated inflammation and protease responses.

PLTP activity and gene expression can be regulated by many factors, including a high-fat-cholesterol diet, LPS injection^35^, glucose^36^, insulin^37^ and diacylglyceride^38^. The PLTP promoter contains farnesoid X-activated receptor (FXR), liver X receptor (LXR) and peroxisome proliferator-activated receptor (PPAR) binding motifs and show five consensus sequences for the transcription factors Sp1 and AP2 required for PLTP transcription^39,40^. Hypoxia induces PLTP gene expression in lung cells^24^, but PLTP activity is reduced in COPD airways due to proteolytic cleavage by serine proteases^15^. Protease release is elevated in COPD lungs but become exaggerated at the time of an exacerbation. Upon infection, lung protease activity is elevated and proteins required for microbial clearance and normal lung function are subjected to enhanced degradation^41^. This is further relevant to this study, as PLTP prevents bacterial growth observed in sepsis^42^ and PLTP activity has a strong correlation with lung function in COPD subjects^15^. Here, we show that the PiZZ form of AAT is less efficient at preventing PLTP cleavage than the PiMM form and PLTP has important anti-inflammatory properties against neutrophils from both AAT deficient and competent individuals. Loss of PLTP expression heightens neutrophil superoxide production and degranulation upon stimulation. Therefore, PLTP expression and activity can be modulated by multiple factors but the disease lung microenvironment in AAT deficiency is not favorable for PLTP integrity and activity.

Neutrophils play a critical role in the host defense, but enhanced activation can significantly contribute to tissue damage during autoimmune and inflammatory diseases, such as AAT deficiency. Determining how PLTP and AAT keep the neutrophil in a basal state is critical to understanding the elevated inflammation observed in AAT deficient subjects. Neutrophils do generate and release AAT that could contribute to protease inhibition, with AAT from deficient individuals less efficient at inhibiting proteases^43^. This may also contribute to neutrophil degranulation, as neutrophils from AAT deficient subjects degranulation at a higher frequency than neutrophils from AAT competent subjects^13^. It was surprising that PLTP and AAT appear to have overlapping roles in preventing inflammation, with both proteins targeting Hck kinase activity. However, AAT also inhibited the activities of two other Src kinases family members, Fgr and Lyn, which may further enhance its anti-inflammatory abilities. Neutrophils from Hck^−/−^Fgr^−/−^ animals have reduced degranulation^44^ and fail to activate a respiratory burst and display reduced F-actin polymerization upon fMLP stimuli^31^. Lyn, Fgr and Hck deficiency has no effect on leukocyte migration but do significantly contribute to inflammation via immune cell activation^32^. Importantly, constitutive activation of Hck in mice leads to the development of an emphysema like phenotype with some pulmonary fibrosis with inflammation that altered lung function and respiratory distress in older animals^45^. Despite fibrosis being rarely documented in AAT deficient subjects, there are reported cases of AAT deficient patients being more susceptible to interstitial lung diseases^46^. Whether PLTP modulates leukocyte migration requires exploring. It should be noted that PLTP and AAT could also interact with multiple other signaling to minimize neutrophil responses, such as G-protein-coupled chemokine/chemoattractant receptors, Fc-receptors, adhesion receptors, TLRs and C-type lectins. These and other potential pathways, in terms of PLTP and AAT signaling, need to be examined in future studies.

Our group have previously demonstrated that saracatinib treatment had protective effects in animal and cell models of cigarette smoke induced COPD^47^. High dosing of saracatinib has been reported to yield several cases of neutropenia in cancer patients^48^. Dasatinib is reported not to affect migration, phagocytosis or killing of bacteria by neutrophils^49^. However, dasatinib does prevent adhesion of human neutrophils in the presence of whole serum^49^ and also prevents TLR ligand induction of TNF-α signaling^50^. Therefore, inhibition of Src family kinases could prevent unnecessary inflammation that contributes to tissue damage, without reducing microbial clearance. However, further studies are required to determine the effect of Src inhibitors on additional neutrophil functions.

There are several proteins that bind to PLTP, including ABCA1, apoA1 and apoA2. Similar to PLTP, ABCA1 and apoA1 both have anti-inflammation abilities^51^. ABCA1 is a cell membrane protein that exports excess cholesterol from cells to apoAI, the major protein in HDLs. The interaction of apoAI with ABCA1 suppresses LPS-induced IL-1β, IL-6, and TNF-α^51^. Importantly, PLTP regulates the size and composition of HDL in the circulation and plays an important role in controlling plasma HDL levels^52^. AAT is also present in HDL^53^ but little is known about the role of AAT in HDL and the subsequent role of PLTP on the circulation of AAT in HDL. Inflammation reduces the concentration of HDL and possibly compromises its function^54^. AAT presence in HDL impacts on inflammation and emphysema formation^55^. Interestingly AAT containing HDL prevents neutrophil and macrophage infiltration into the airways and reduces elastase-induced production of IL-6, MCP-1, TNF-α, MMP-2 and MMP-9^55^. However due to the interactions of PLTP with ABCA1 and HDL, and the protective role of AAT on PLTP, investigating PLTP binding proteins would be of great interest. ApoA1 and ABCA1 has also be implicated in mediating neutrophil inflammation in asthma^56^ and ApoA1 decreases neutrophil degranulation and superoxide production^57^. Therefore, ABCA1 and ApoA1 could interact with PLTP and AAT to influence their anti-inflammatory potential.

There are several limitations that need to be discussed here. Firstly, we compare changes in human and mouse neutrophil degranulation. The frequency of neutrophils in mice is less compared to humans. Mice have many different receptors and several of their granule content differ to humans^58^. Mice do not express FcαRI^59^, which plays several important roles in human neutrophils, such as oxidative burst, cytokine release, and phagocytosis. Therefore, it is important to consider the possibility that the neutrophil degranulation responses may not occur in precisely the same way in humans and mice. Recently, loss of the mouse *Serapina1* genes was shown to result in age dependent emphysema in a similar manner to the human disease^60^, which suggests that the mouse still represents a useful model for AAT deficiency. Secondly, we primarily focus on neutrophil degranulation and not on other neutrophil functions. The role of PLTP on chemotaxis, NETosis and phagocytosis needs to be addressed in future studies. Third, the role of Hck and other Src kinases in AAT deficiency requires further investigating prior to exploring the potential of these inhibitors in the human disease. Fourth, we use higher biological levels of PLTP (0.5 µM versus 0.23 μM)^61^ to treat neutrophils, as the rPLTP utilized here for the human samples is less active than corresponding concentrations of PLTP activity in plasma (Supplementary Fig. S1). The AAT levels used here (2 µM) are lower than plasma (ranging from 20–39 µM) but within range of lung concentrations (2 µM). Therefore, non-specific effects may be observed with higher total PLTP levels. Fifth, most of the patient population were former smokers, which can alter neutrophil degranulation, inactivate AAT^62^ and lead to PLTP inactivation. At the time of blood collect, no patient was an active smoker (Table 1). Finally, the only difference observed for AAT and PLTP signaling mediating neutrophil degranulation was AAT inhibition of Fgr and Lyn.

**Table 1:** Demographics of Study Subjects.

	PiMM	PiZZ	PiZZ + AAT
Number	22	22	8
Age (years)	57.2 ± 4.2	58.5 ± 2.2	60 ± 8.7
Gender (Male/Female)	13/9	13/9	6/2
AAT genotype (MM/ZZ/ZS)	22/0/0	0/21/1*	0/7/1*
Baseline plasma AAT (μM)	24.4 ± 2.6	4.9 ± 1.7*	12.1 ± 2.3*
Former smokers (%)	0	59.1*	62.5*
Current smokers (%)	0	0	0
FEV1% Predicted	100.2 ± 4.6	56.0 ± 3.9*	50.9 ± 10.1*
FVC % Predicted	96.4 ± 3.3	74.6 ± 5.7*	88.4 ± 14.1*
FEV1/FVC %	81.65 ± 6.4	50.4 ± 4.1*	44.8 ± 5.7*
DLCO % Predicted	102.7 ± 6.3	64.3 ± 3.9*	57.0 ± 14.4*

^*^Denotes a p < 0.05 when comparing to PiMM group.

In conclusion, PLTP activity diminishes neutrophil activation and degranulation and, similar to AAT, PLTP reduces Hck activity in neutrophils. Reduced PLTP activity in the AAT deficient lungs could leave the lung more susceptible to neutrophil associated inflammation and worsening of disease progression.

## Methods

### Human samples

BALF was obtained from healthy never smokers with a normal AAT (n = 8) and AAT deficient patients (n = 22) (see Table 1 for demographics). All subjects were free from exacerbation for six months prior to this study. A subset of the PiZZ subjects (n = 8) were receiving AAT augmentation therapy, Zemaira^R^ intravenously, at standard doses (60 mg/kg/week). Blood was drawn and BALF collected^63^. AAT deficiency was determined, using the Grifols AlphaKit test kits processed at the Alpha-1 Antitrypsin Genetics Laboratory at GeneAidyx LLC (Alachua, Florida), by assessing low serum levels, followed by genotyping, phenotyping and sequencing. Written and informed consent was obtained from all study participants and the trial was approved by the institutional review board of the University of Miami School of Medicine, Mount Sinai Icahn School of Medicine and State University of New York Downstate Medical Center. All methods were performed in accordance with the institutional review board guidelines and regulations.

### PLTP activity and degradation analysis

PLTP activity was measured by using an assay kit (Roar Biomedical, New York, NY), as previously described^64^. Activity was standardized to BALF urea concentrations determined by a commercially available kit (Abnova, Walnut, CA). BALF was concentrated utilizing 3,000 NMWL Centriplus filter devices (Millipore) and PLTP immunoblots were performed to observe PLTP degradation utilizing a rabbit anti-human PLTP polyclonal rabbit antibody (Cat. # NB400-106, Novus Biological, Littleton, CO, USA) that recognizes a partial peptide of human PLTP. Recombinant human PLTP (Novus Biological) was incubated with 1 μM AAT protein (from either a PiMM or PiZZ subject) for one- hour at 37 °C, followed by the addition of 10 μl of BALF (PiZZ subjects) in PBS to a final volume of 20 μl for 24 hours at 37 °C. PLTP degradation was examined by immunoblotting samples and detection with a PLTP antibody.

### Isolation of AAT protein from PiMM and PiZZ subjects

Blood was collected from PiMM or PiZZ individuals and AAT was isolated from the serum of AAT deficient (PiZZ) and competent nonsmoker individuals (PiMM) by affinity chromatography using AAT select resin (Cat # 17547201, GE Healthcare, Piscataway, NJ) as previously described^6^. Briefly, serum was incubated with pre-clear 4% crosslinked beaded agarose resin (Cat # 26150, Thermo Fisher Scientific, Waltham, MA) overnight at 4 °C. The flow through was incubated with AAT select resin for 24 hours at 4 °C. The resin underwent 11 washes with 20 mM Tris, 150 mM NaCl, pH 7.4. AAT was eluted from the resin with 20 mM Tris, 2 M MgCl_2_, pH 7.4. Elutions underwent dialysis against 20 mM Tris, pH 7.4. Following elution, AAT levels were quantified and stored at −80 °C until required. AAT antiprotease activity was determined by incubating AAT samples with NE at a 2:1 molar ratio for 30 minutes at 37 °C with NE and using the ENZO Neutrophil elastase colorimetric drug discovery kit, as recommended by manufacturers. Activity was recorded as a change in absorbance at 405 nm.

### Neutrophil isolation

Neutrophils were isolated from venous peripheral blood obtained from AAT competent (PiMM) and deficient (PiZZ) volunteers. Density gradient centrifugation was conducted in LymphoPrep (Axis-Shield PoC AS, Oslo, Norway) to isolate the red cell pellet containing the neutrophil population. Neutrophils were further purified as described previously^12^. Neutrophils were also isolated from the bone marrow of mice as previously described^6^. Male and female adult C57BL/6J and *Pltp*^−/−^ mice^65^, were maintained in a specific pathogen-free facility at SUNY Downstate Medical Centre. Neutrophils were placed in PBS supplemented with 4 mM glucose prior to stimulation. Cells were treated with either 2 μM PiMM or PiZZ AAT, 0.5 μM PLTP, 100 nM Saracatinib (LC Laboratories) or 100 nM dasatinib for 10 minutes prior to LTB_4_ or fMLP stimulus. Secreted protein and whole cell protein were collected at several time points. All animal experiments were performed with approval from SUNY Downstate’s Institutional Animal Care and Use Committee. This study was performed in strict accordance with the recommendations in the Guide for the Care and Use of Laboratory Animals of the National Institutes of Health and Institutional Animal Care and Use Committee (IACUC) guidelines and according to the Declaration of Helsinki conventions for the use and care of animals.

### Neutrophil degranulation and superoxide production determination

Secreted protein samples from purified neutrophils (2 × 10^7^ cells/ml) were examined by ELISA, activity assays and immunoblots. NE levels were determined by ELISA (R&D Systems). Cathepsin G activity was determined in samples with a colorimetric cathepsin G activity assay kit (Abcam, ab126780, Cambridge, MA), as described by manufacturers. Immunoblots were performed with anti-human lactoferrin polyclonal rabbit (immunogen was full length native protein, Cat. # ab15811, Abcam) and anti-human hCAP18 rabbit polyclonal antibodies (Cat. # PA5-20513, Invitrogen). BALF gelatinase activity (MMP9) was determined by gelatin zymography^66^. Densitometry was performed on MMP9 positive bands and represented densitometry units (DU) measured by pixel intensity of bands, using Bio-Rad Laboratories Image Lab software (version 4.0, build 16). Equal cell numbers in each experiment was demonstrated by profiling electrophoretic of whole cell lysates in a Coomassie blue stained gel, i.e. loading control. O_2_^−^ production was quantified by O_2_^−^ dismutase–inhibitable reduction of cytochrome c at 550 nm, as previously described^67^.

### Intracellular signaling

Protein was isolated from neutrophils using lysis buffer (10 mM HEPES (pH 7.9), 1.5 mM MgCl_2_, 10 mM KCl, 0.5 mM PMSF, 0.5 mM DTT, 0.2% Igepal CA-630) and sonication. Immunoblots were conducted on the protein from cells to determine levels of phosphorylated p38(Thr180/Tyr182) (anti-human phosphorylated p38(Thr180/Tyr182) polyclonal rabbit antibody against a synthetic phosphopeptide corresponding to residues surrounding Thr180/Tyr182 of human p38 MAPK; Cat. # 4511), Src(Tyr416) (anti-human polyclonal rabbit antibody against a synthetic phosphopeptide corresponding to residues surrounding Tyr419 of human Src; Cat # 2101) and total protein levels of Hck (anti-human monoclonal rabbit antibody against a synthetic peptide corresponding to residues surrounding Lys60 of human Hck protein; Cat. # 14643), Fgr (anti-human Fgr polyclonal rabbit antibody against a synthetic peptide corresponding to residues close to the amino terminus of human Fgr; Cat. #2755), Lyn (anti-human LIF polyclonal rabbit antibody from the epitope mapping at the N-terminus of Lyn of human origin; Cat. # SC-15, Santa Cruz Biotechnologies), p38 (anti-human p38 polyclonal rabbit antibody against a synthetic peptide corresponding to the sequence of human p38 MAPK; Cat. # 9212), and β-actin (anti-human β-actin polyclonal rabbit antibody against a synthetic peptide corresponding to amino-terminal residues of human β-actin; Cat. # 4967 unless specified all antibodies were from Cell Signaling, Danvers, MA). Uncropped immunoblots are present in Supplementary Figs 2–6. Src kinase activity was determined as previously described^47^ utilizing a tyrosine kinase activity kit (MK410; Takara Bio, Mountain View, CA). Hck, Fgr and Lyn were immune-precipitated from neutrophil protein samples and tyrosine kinase activity was determined as instructed by manufacturers in the tyrosine kinase kit. A positive protein tyrosine kinase control is supplied in this kit. Results are presented as relative activity compared with non-treated wild-type neutrophil protein samples. Immunoblots were performed to confirm positive immune-precipitation.

### Statistical analyses

Data are expressed as dot plots and bar graphs with the means ± S.E.M highlighted in red. Differences between two groups were compared by Student’s t test (two-tailed). Experiments with more than 2 groups were analyzed by ANOVA with Tukey’s post hoc test analysis. p values for significance were set at 0.05 and all significant changes were noted with *. All analysis was performed using GraphPad Prism Software (Version 6.0 h for Mac OS X).

## Electronic supplementary material

Supplementary information
