# Supplementary material for: Phospholipid transfer protein and alpha-1 antitrypsin regulate Hck kinase activity during neutrophil degranulation
Source: Sci Rep. 2018 Oct 18;8:15394. doi: 10.1038/s41598-018-33851-8 (PMC6193999; doi:10.1038/s41598-018-33851-8)
Supplement: Supplementary file 1 — Supplementary information [file 41598_2018_33851_MOESM1_ESM.pdf]

Supplementary Information

Phospholipid transfer protein and alpha-1 antitrypsin regulate Hck kinase activity during neutrophil degranulation

Pius Ochieng[1], Sridesh Nath[2], Reane Macarulay[3], Edward Eden[4], Abdoulaye Dabo[2,5], Michael Campos[6], Xian-Cheng Jiang[5], Robert F. Foronjy[2,5] and Patrick Geraghty[2,5*]

[1]Pulmonary, Critical Care and Sleep Division, Geisinger Medical Center, Danville, PA 17822, USA; [2]Department of Medicine, State University of New York Downstate Medical Center, Brooklyn, NY 11203, USA; [3]Trinity College, Dublin 2, Ireland; [4]Division of Pulmonary and Critical Care Medicine, Mount Sinai St Luke's Hospital, New York, NY 10025, USA; [5]Department of Cell Biology, State University of New York Downstate Medical Center, Brooklyn, NY 11203, USA; [6]University of Miami Miller School of Medicine, Miami, Florida, FL 33136, USA.

*Corresponding author:

Patrick Geraghty, PhD

Departments of Medicine and Cell Biology,

State University of New York Downstate Medical Center,

450 Clarkson Avenue, Room 2-10, MSC-5

Brooklyn, NY 11203,

USA

Phone: 718 270 3141

Email: Patrick.Geraghty@downstate.edu

Supplementary Methods

Plasma and recombinant PLTP comparison

PLTP levels were determined by ELISA in plasma, using the LSBio PLTP ELISA Kit (Cat. # LS-F22111, Seattle, WA, USA). PLTP activity assays were performed on 5 ul of saline containing 20 pg of PLTP from plasma, determined by ELISA, or recombinant PLTP (rPLTP) by using the Roar Biomedical PLTP activity kit. PLTP activity was expressed as pM/20 pg PLTP protein over 1 hour.

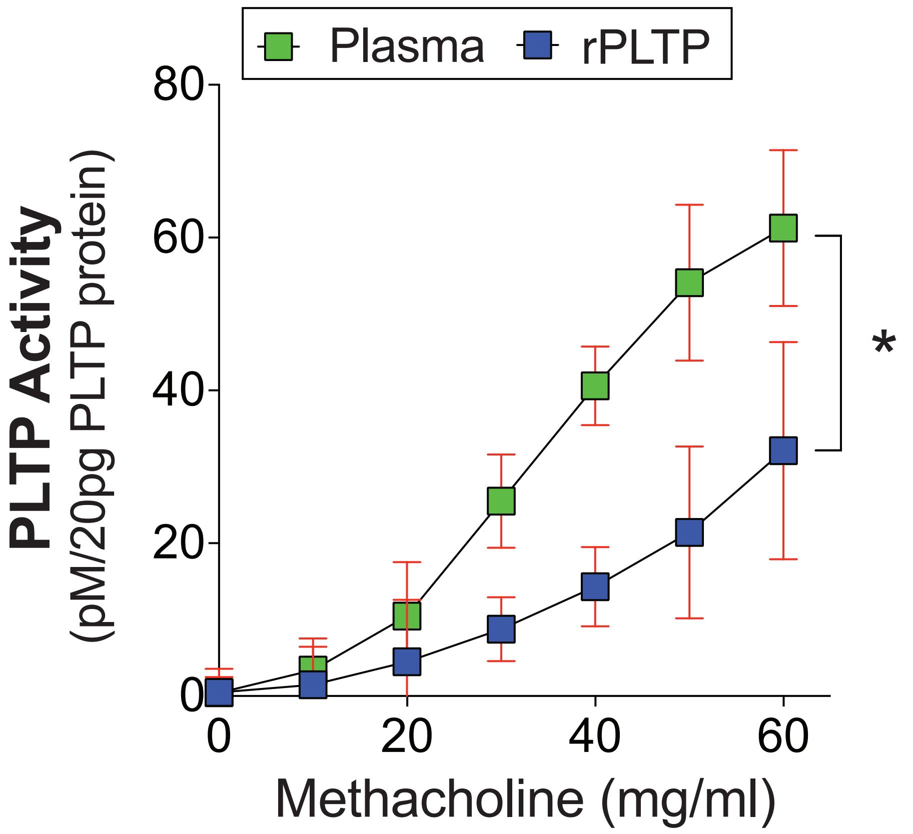

Supplementary Figure 1. Recombinant PLTP has less activity than PLTP in plasma. PLTP activity was performed on 20pg PLTP from plasma or rPLTP. PLTP activity was expressed as pM/20 pg PLTP protein over 1 hour. Graphs are represented as mean ± S.E.M. * denotes a p value <0.05, when comparing both treatments connected by a line, determined by 2-way ANOVA with Tukey's post hoc test.

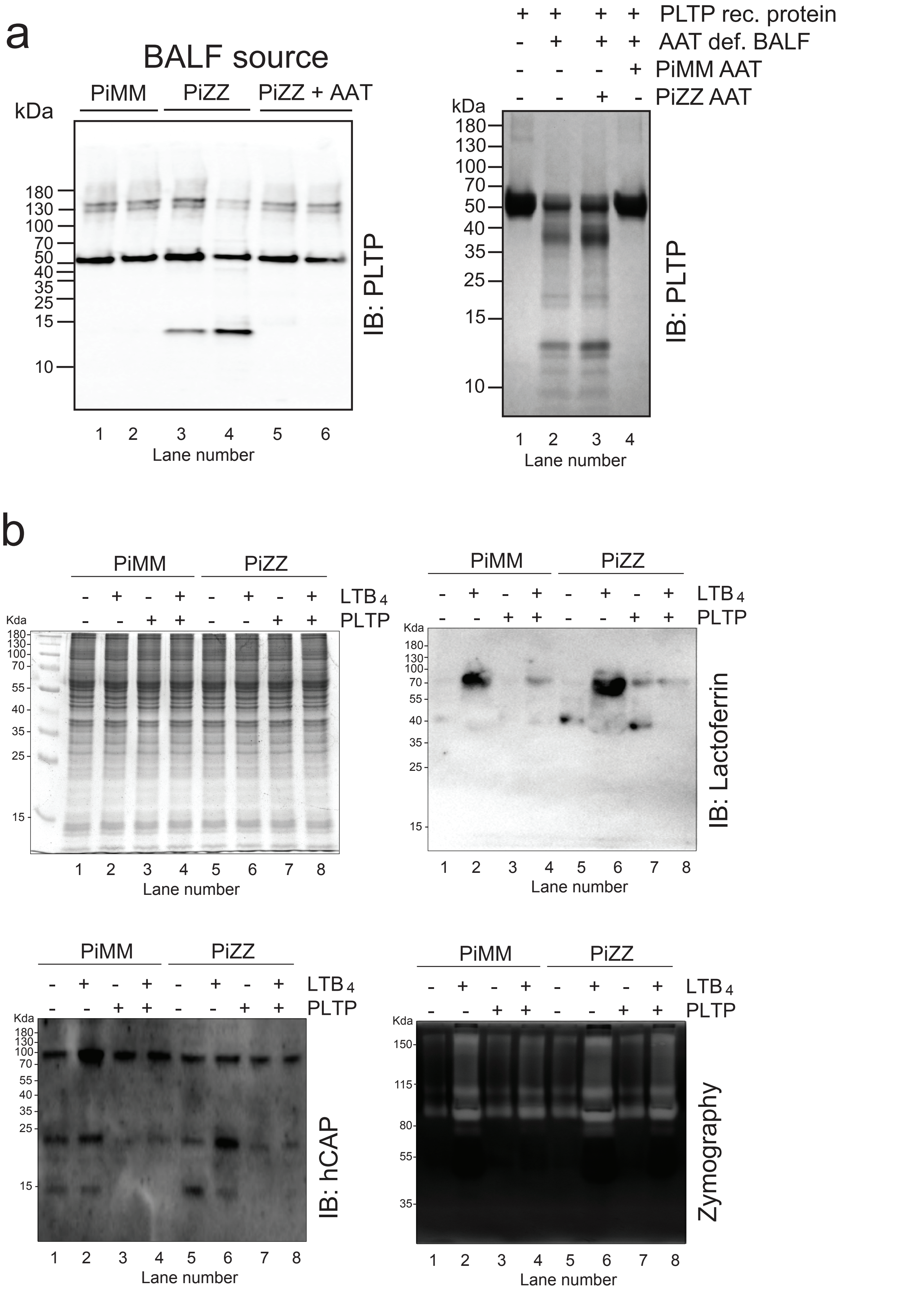

Supplementary Figure 2. Non-cropped pictures of blots. Non-cropped picture of immunoblots and zymogram gel presented in Figures (a) 1 and (b) 2.

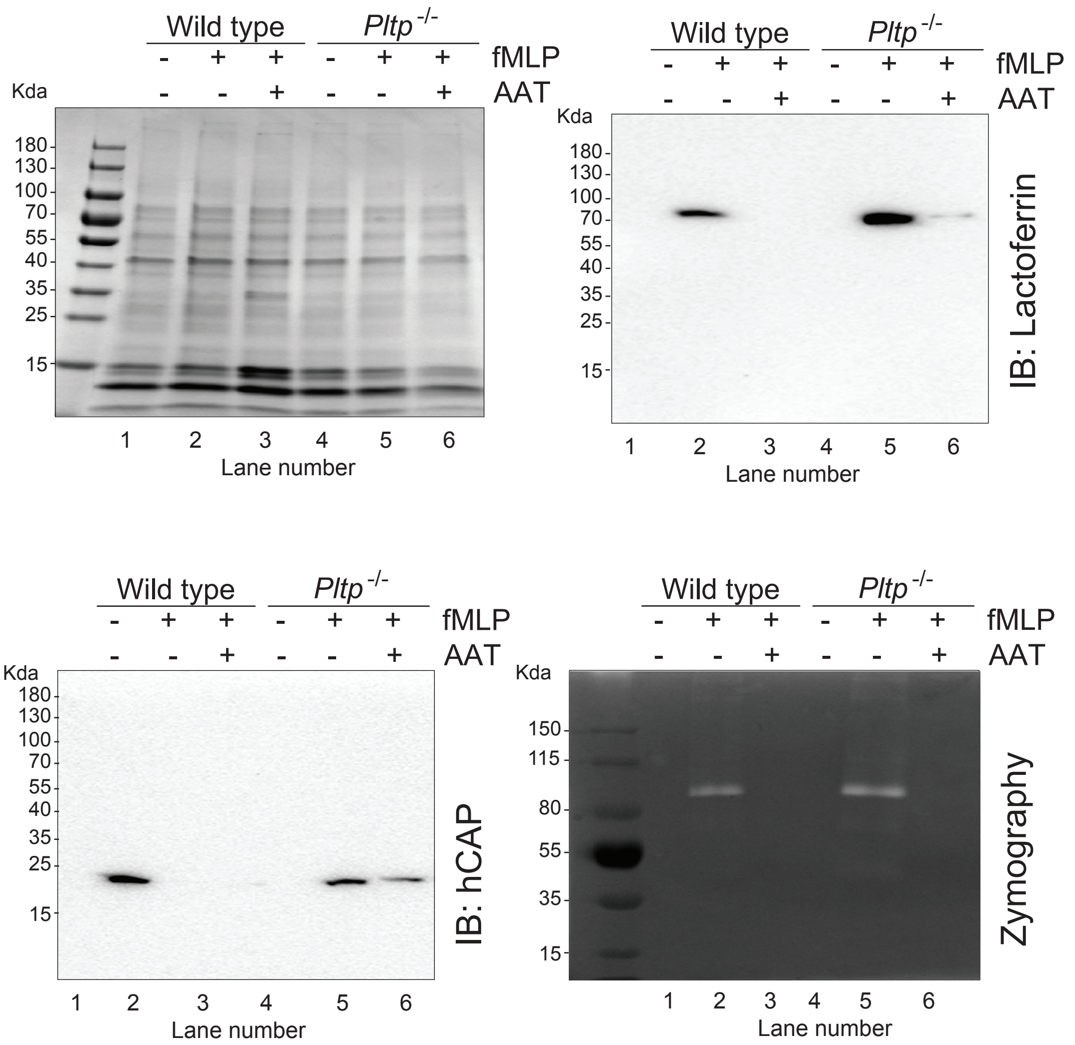

Supplemental Figure 3. Non-cropped pictures of blots. Non-cropped picture of immunoblots and zymogram gel presented in Figure 3.

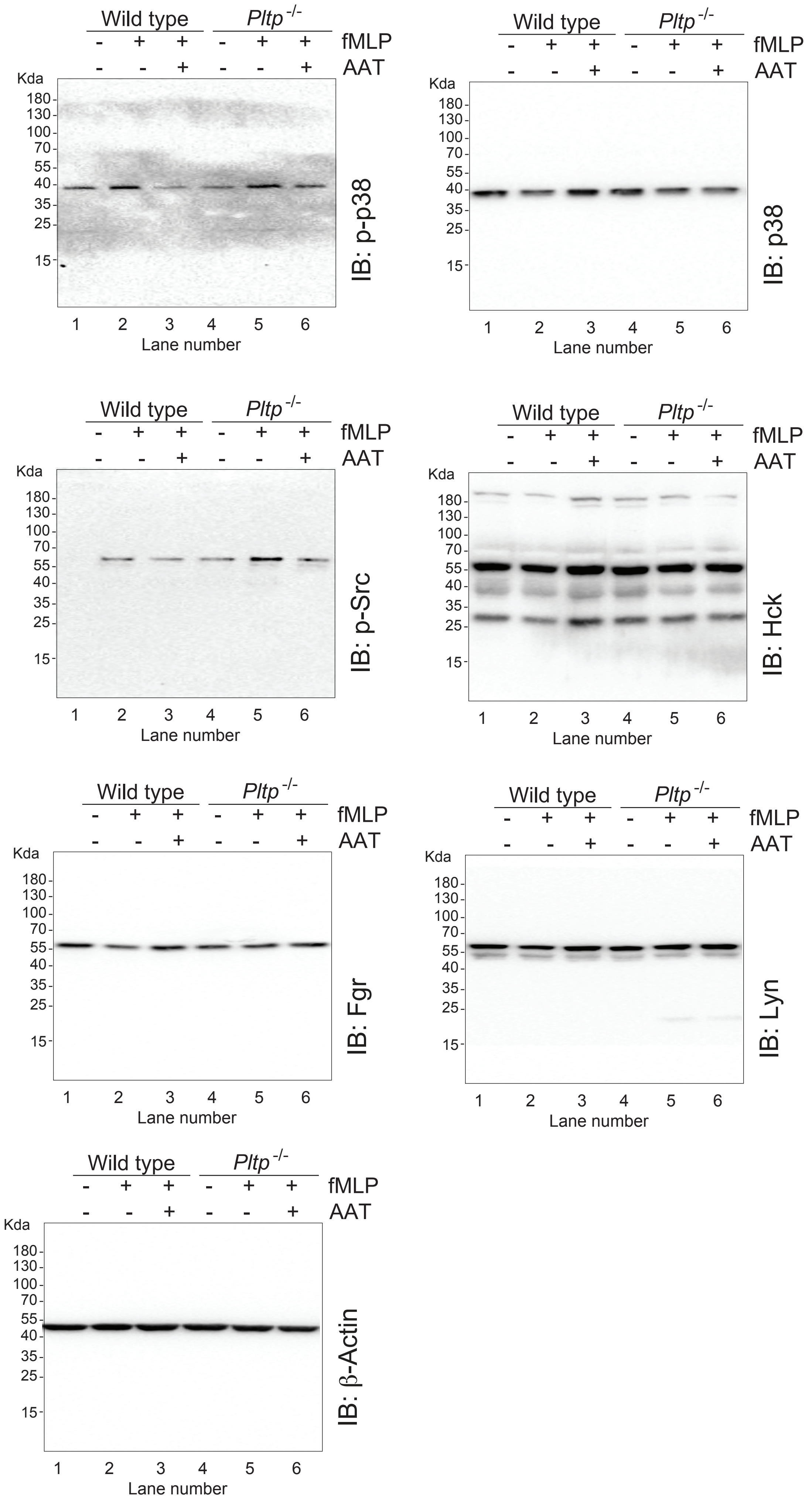

Supplemental Figure 4. Non-cropped pictures of blots. Non-cropped picture of immunoblots presented in Figure 4.

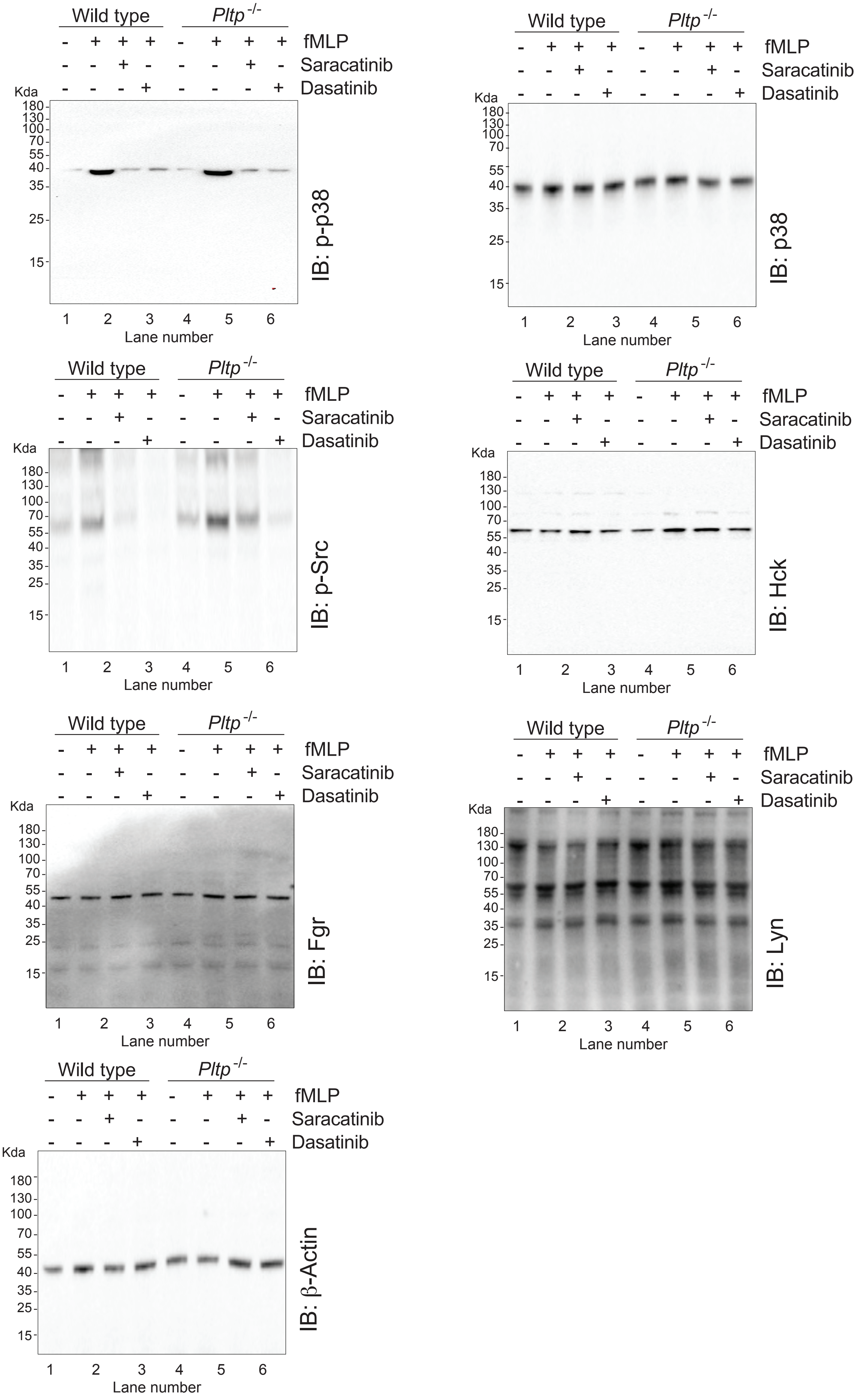

Supplemental Figure 5. Non-cropped pictures of blots. Non-cropped picture of immunoblots presented in Figure 5A.

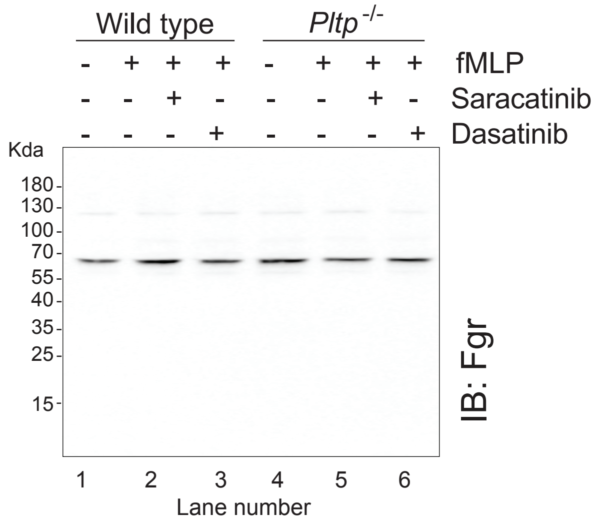

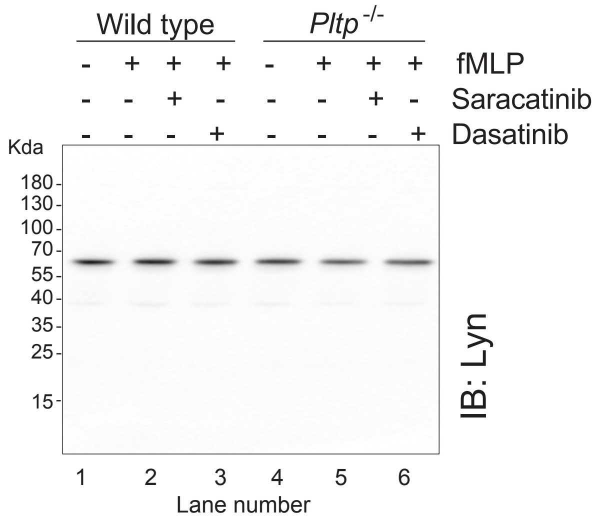

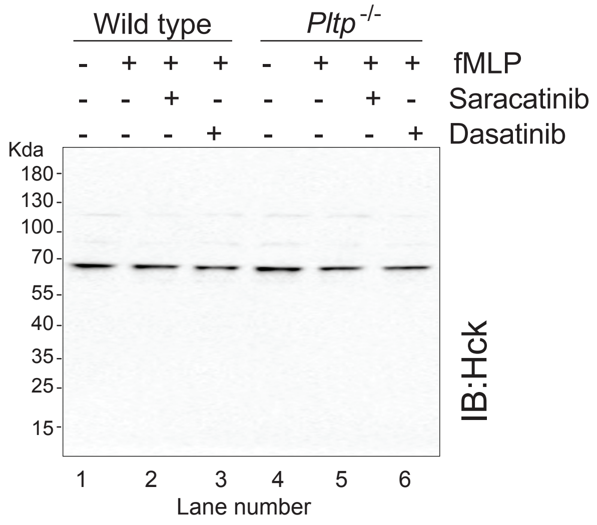

Supplemental Figure 6. Non-cropped pictures of blots. Non-cropped picture of immunoblots presented in Figure 5B.
